# Congenital infantile hypertrophic pyloric stenosis in preterm dizygotic twins infants diagnosed early: A case report

**DOI:** 10.1016/j.ijscr.2023.109069

**Published:** 2023-11-17

**Authors:** Mundeke Mujinya Bienfait, Buhoro Baabo Gisèle, Maunga Vangi Annie, Safari Kibanja Anderson, Baanitse Munihire Jeannot, Joshua Muhumuza

**Affiliations:** a Charité Maternelle general Hospital of Goma, Goma, Democratic Republic of the Congo; b Faculty of Medicine, Université Catholique la Sapientia de Goma, Goma, Democratic Republic of the Congo; c Faculty of Medicine, Université de Goma, Goma, Democratic Republic of the Congo; d Faculty of Clinical Medicine and Dentistry, Department of Surgery, Kampala International University Western Campus, Ishaka, Uganda

**Keywords:** Hypertrophic pyloric stenosis, Preterm dizygotic twin, Early diagnosis, Case report

## Abstract

**Introduction and importance:**

The association in the occurrence of hypertrophic pyloric stenosis (HPS) is 0.25 % to 0.44 % between monozygotic twins and 0.05 % to 0.10 % in dizygotic twins. A combination of genetic and environmental factors may have contributed to the occurrence of HPS. In view of the few related cases reported recently, we present two dizygotic twins who were diagnosed with HPS.

**Case presentation:**

This report describes a rare case of congenital infantile hypertrophic pyloric stenosis in preterm dizygotic twins diagnosed early, in which the first case presented with severe clinical features and managed surgically while the second presented with moderate features and hence managed non-operatively with atropine for 14 days. At 6 months of age, both twins continued to tolerate feeds, demonstrated satisfactory weight gain and had achieved appropriate developmental milestones. The postoperative course was uneventful in the twin A.

**Clinical discussion:**

Congenital HPS in premature twins remains an underdiagnosed pathology due to its clinical picture mimicking digestive intolerance to feeds. The mean age at diagnosis is about 38 days, and only 0.4 % of all children suffering from HPS show symptoms in the first 3 days of life. Symptom relief is achieved after a classic pyloromyotomy is performed by a more preferable laparoscopic technique or using the open surgical technique.

**Conclusion:**

If one of the dizygotic twins has HPS, the other baby should be evaluated for the same diagnosis as early as possible, to ensure timely management. HPS with moderate clinical features can be treated with atropine for 14 days while severe HPS should be treated by pyloromyotomy.

## Highlights

• We report a case of congenital infantile hypertrophic pyloric stenosis in preterm dizygotic twins infants diagnosed early.
• A palpable pyloric mass was found in the epigastric region.
• Ultrasound performed found a s stenosis with antral nipple sign suggesting a hypertrophic pyloric.
• Ultrasound is an excellent technique to evaluate the hps but remains operator dependent.
• HPS with moderate clinical features can be treated with atropine while severe symptoms require pyloromyotomy.

## 1 Introduction

Infantile hypertrophic pyloric stenosis (IHPS) is a common cause of gastrointestinal obstruction in newborns, but its exact etiology remains unclear [1]. Prior to recognition of this disease as an entity by Hirschsprung in 1888 [2] and description of pyloromyotomy by Ramsteatd in 1911 [3], mortality rates exceeded 50 % [4]. The association of HPS between monozygotic twins is 0.25 % to 0.44 % and in dizygotic twins it is 0.05 % to 0.10 %, making it uncommon in the population [5]. IHPS has an incidence of 20.09 per 10.000 live births [6]. The incidence of HPS in the Caucasian population is 5/1000 newborns compared to the African population in which it is rarer. In the United States of America, the frequency in white children is 0.13 % of the total live births [5].

The onset of symptoms is usually abrupt and dramatic, presenting with non-bilious emesis resulting from hypertrophy and hyperplasia of the pylorus, usually between the second and eighth week of life [7]. A great amount of research has been conducted regarding this disease, but the exact etiology remains unknown. In 1961, the hypothesis of the multifactorial threshold model of inheritance was suggested [8]. In recent years, environmental factors have been associated with IHPS. Children from a smoking mother have a higher risk of IHPS [6]. Pesticides have also been reported as a potential cause of IHPS [9]. A combination of genetic and environmental factors may contribute to the occurrence of HPS. In review of the few similar cases reported recently, we present two dizygotic twins who were diagnosed with HPS [5,6].

The aim of this report was to describe twins with HPS, in which the first case presented with severe clinical features and was managed surgically while the second presented with moderate clinical features and hence managed non-operatively with atropine. The work has been reported in line with the SCARE criteria [10].

## 2 Case presentation

These were dizygotic premature twins born at 33 weeks of amenorrhea; hospitalized in the neonatology unit for 14 days before they were transferred to the pediatric surgery department. They had been admitted to the neonatology unit at the 15th minute of life due to respiratory distress and low birth weight.

They were born to a 26-year-old primiparous mother carrying a monochorionic monoamniotic dizygotic twin pregnancy, with no family history of consanguinity or HPS and to a father aged 29 years. There was no history of fertility drug or contraceptive use. There was no history of smoking nor comorbidity. At 31 weeks, preterm rapture of membranes resulted in oligohydramnios that was confirmed by sonography and preterm labor. Except for dexamethasone and magnesium the mother received following the diagnosis of preterm labor and oligohydramnios, the pregnancy was unremarkable. Both twins were born by caesarean section with the male twin A weighing 1700 g and measuring 41 cm in length, while the female twin B, weighed 1600 g and measured 40 cm in length. The Apgar score was 7/8/9 for the twin A and 5/8/10 for twin B at one, five and ten minutes respectively. No obvious malformations were identified among the twins.

The twins received immediate essential newborn care in accordance to the World Health Organization (WHO) guidelines. Respiratory distress syndrome with a Silverman score of 4/10 in twin A and 5/10 in twin B was noted, with signs of prematurity (Jean-Ballard score of 20). The diagnosis of Hyaline membrane disease and neonatal infection in prematurity was initially made in the twins. Twins A and B were on antibiotics before the diagnosis of HPS (intravenous Cefotaxime) and were fed breast milk alternating with infant formula milk using an oro-gastric tube. Feeding started on the second day for Twin A and the third day for Twin B.

White blood count and C-reactive protein tests were normal for both twins. Twin A: WBC = 15,000/mm^3^, Hemoglobin = 17.5 g/dl, Platelets = 255,000. Twin B: WBC = 13,500/mm^3^, Hemoglobin = 15.4 g/dl, Platelet = 210,000.

**Fig. 1:**
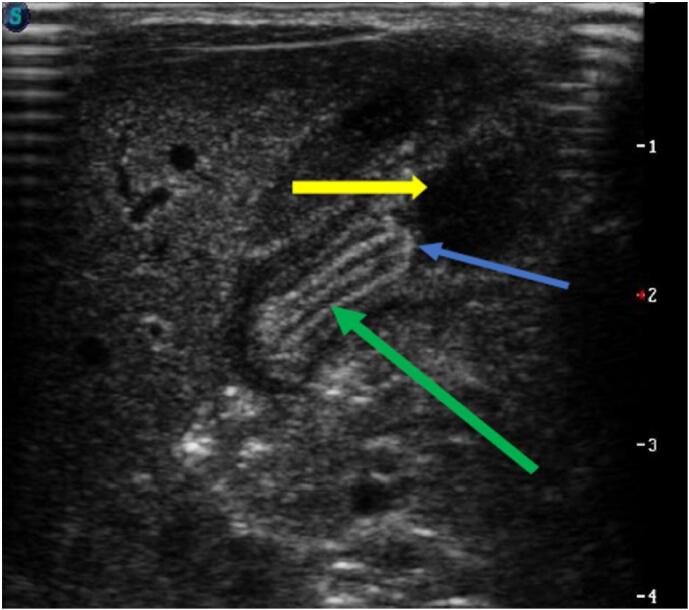
Abdominal ultrasound image of Twin A at 10 days of life, stenosis measuring 5 mm in thickness and 2.1 cm in length (Green arrow) with antral nipple sign (Blue arrow) representing protruding pyloric mucosa and the yellow arrow is the stomach. (For interpretation of the references to colour in this figure legend, the reader is referred to the web version of this article.)

**Fig. 2:**
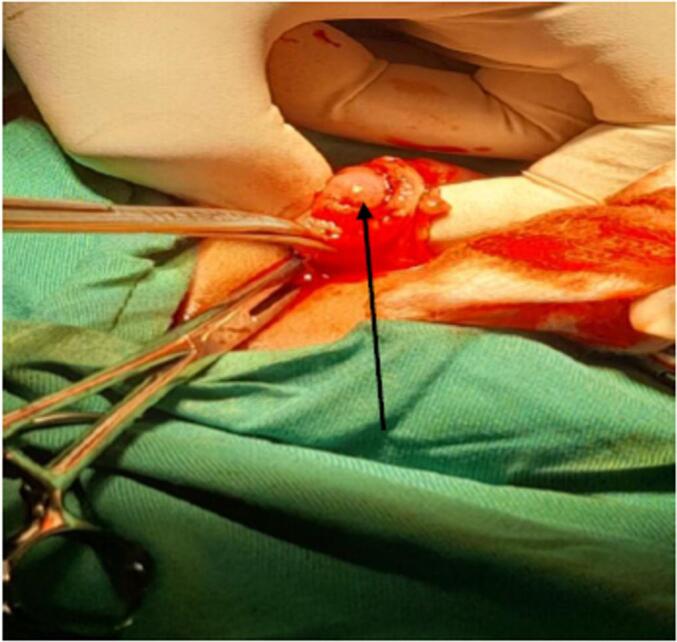
Intraoperative picture, showing correction of a congenital hypertrophic pyloric stenosis by pyloromyotomy (arrow pointing at pyloric mucosa). Twin A underwent an open extra mucosal pylorotomy according to fredt-ramstedt (this figure).

At 9 days of age, twin A developed projectile postprandial non bilious vomiting. On physical examination, weigh was 1550 g and a pyloric mass was palpable. An abdominal ultrasound done on the 10th day of life revealed stenosis measuring 5 mm in thickness and 2.1 cm in length with antral nipple sign representing protruding pyloric mucosa (Fig. 1*)*. Both twins were transferred to the pediatric surgery department on the 13th day of life for surgical management. Twin A underwent an open extra mucosal pylorotomy according to fredet-ramstedt (Fig. 2).

At 12 h postoperatively, a gastrograph meal (water-soluble nonionic gastrointestinal radiopaque agent) was done. A passage of the contrast agent was seen up to the duodenum, confirming patency. Postoperatively, feeds were initiated at 24 h post-surgery and were well tolerated. The infant was discharged on the 3rd postoperative day.

Twin B presented with moderate postprandial vomiting at 12 days of life. There was a small palpable pyloric mass on physical examination and stenosis was seen on sonography. The arterial blood gas findings for twin B were: pH = 7.38, PaO_2_ = 90 mmHg, PaCO2 = 35 mmHg. Twin B was initially placed under observation of clinical features and treated with atropine before each meal, with a plan for surgery if symptoms persisted or became worse. In the end, the evolution was good and he did not undergo surgery since the hypertrophy of his pylorus was small. Atropine was given for 14 days under observation, and the post prandial vomiting reduced from 7 times to 1 time a day in the 2 weeks.

### 2.1 Follow up

At 3 months of life, twins A weighed 4800 g while twin B weighed 3210 g. No complaints were reported in either twin. At 6 months, both twins continued to tolerate the feeds, demonstrated satisfactory weight gain and achieved developmental milestones. No complications were observed in both babies.

## 3 Discussions

The incidence of HPS is higher in non-Hispanic white males, firstborns, preterm births (<37 weeks) and infants from multiple gestations [5,6]. There is, however, a variation in the incidence of HPS in the Africans, which varied from 1/5500 to 12.9/10,000 children. The reported incidence of 1/5500 live births in Tanzania was considerably lower than trends seen in other parts of the world [1].

An early onset of HPS presenting soon after birth as was seen in these twins has only scarcely been reported. Demian et al. in a large case–control study revealed that, only 6 % of infants diagnosed with HPS were <14 days old [11]. These infants had a significantly higher positive family history for HPS when compared to infants who presented with HPS after 14 days of life. Besides the early presentation of HPS, also the late presentation of HPS has been reported in the literature as a rare event [12].

In our report, two dizygotic twins who developed HPS are described, which is interesting because Icnoti et al. (2022) in Mexico noted similar cases [5]. HPS has also been reported in dizygotic twins simultaneously [13]. The mean age at diagnosis is about 38 days, and solely 0.4 % of all children suffering from HPS show symptoms in the first 3 days of life [14]. Furthermore, a decreased risk of developing HPS has been shown with increased maternal age and the number of pregnancies [4], and it is very uncommon for preterm infants to develop signs of HPS in the first week of life. Though the twins in this report were born pre term, the mother was only 26 years old and this was her first pregnancy.

Even if preterm infants may not show the typical symptoms of HPS, such as projectile vomiting and metabolic alkalosis, mild symptoms such as regurgitation are reported to occur in the first days of life in 2/3 of all cases [13]. HPS has also been reported to occur in triplets and dizygotic twins simultaneously [13,15], which was the case in the present report. Darlene in 2018 stated that when one twin has HPS, about 80–90 % of the time the other twin also “presents with it” [16].

It is very rare for premature infants to develop signs of HPS during the first week of life [17]. In the present study, the age of onset of signs was 10 days of life. The usual clinical picture of HPS is projectile vomiting, and in expert hands the enlarged pyloric muscle can be palpated as an olive in the abdomen. [5], which was the case in our observation. Standard ultrasound criteria for measurement of pyloric muscle size in children with HPS may be valid for confirmation of the diagnosis of congenital HPS [17].

Symptom relief is achieved after a classic pyloromyotomy is performed by a more preferable laparoscopic technique or using the open surgical technique [13]. A medical treatment based on atropine has been tested by other teams intravenously until the vomiting has stopped, then orally before each feed; as is the case with twin B in our report. This treatment improves digestive tolerance during the natural evolution of the pathology [18]. Though twin B was treated with atropine, the resolution of symptoms was slower, which in turn was associated with prolonged hospital stay. In settings where surgeons are much fewer than recommended and anesthesia specialists for neonates not available, management with atropine may be a viable option.

We treated twin A by supra-umbilical midline laparotomy of about 3 cm, we visualized a hypertrophic stenosis of the pylorus and we performed an extra mucosal pyloromyotomy in an avascular zone according to fredet-Ramstedt technique and the outcome was uneventful. A gastrographin meal was done which confirmed digestive outlet continuity on the 1st postoperative day.

## 4 Conclusion

Congenital HPS in premature twins remains an underdiagnosed pathology due to its clinical picture mimicking gastroesophageal reflux.

If one of the dizygotic twins has HPS, the other baby should also be evaluated for it, as early as possible, to ensure timely management. HPS with moderate clinical features can be treated with atropine while severe symptoms require pyloromyotomy.

## Consent for publication

Written informed consent was obtained from the patient's parents/legal guardian for publication and any accompanying images. A copy of the written consent is available for review by the Editor-in-Chief of this journal on request.

## Provenance and peer review

Not commissioned, externally peer-reviewed.

## Authorship

All authors attest that they meet the current ICMJE criteria for Authorship.

## Ethical approval

Not applicable.

## Funding

There was no external funding sourced for this report.

## Author contribution

MMB, BBG, MVA and SKA managed the patient and wrote the first draft. BMJ and JM helped in editing and reviewing the paper. All authors read and approved the final version to be published.

## Guarantor

Baanitse Munihire Jeannot.

## Research registration number

Not applicable.

## Conflict of interest statement

The authors declare no conflict of interest.
